# Factors associated with intention of clinical pharmacists and candidates to provide pharmaceutical care: application of theory planned behaviour

**DOI:** 10.1186/s12909-023-04658-7

**Published:** 2023-09-20

**Authors:** Kamer Tecen-Yucel, Nesligul Ozdemir, Emre Kara, Kutay Demirkan, Mesut Sancar, Betul Okuyan

**Affiliations:** 1 Department of Clinical Pharmacy, Anadolu University, Faculty of Pharmacy, 26210 Eskisehir, Türkiye; 2 Department of Clinical Pharmacy, Inönü University, Faculty of Pharmacy, Malatya, Türkiye; 3 Department of Clinical Pharmacy, Hacettepe University, Faculty of Pharmacy, Ankara, Türkiye; 4 Department of Clinical Pharmacy, Marmara University, Faculty of Pharmacy, Istanbul, Türkiye

**Keywords:** The theory of planned behaviour, Pharmaceutical care, Clinical pharmacy

## Abstract

**Background:**

Postgraduate education programs in clinical pharmacy have become widespread in Türkiye. This study aimed to identify factors associated with the intention of Turkish clinical pharmacists and candidates (who were graduates and students of postgraduate clinical pharmacy programs) to provide pharmaceutical care.

**Methods:**

This prospective observational study was conducted between June 2021 and May 2022. After searching relevant studies, an expert panel discussion, translation, cultural adaptation, and a pilot study developed a 52-item Turkish scale based on the Theory of Planned Behavior (TBP). Cronbach alpha for each construct was calculated after an explanatory factor and test–retest reliability analysis. An online survey link was sent to all graduates or candidates of postgraduate clinical pharmacy programs in Türkiye. After univariate regression analysis, the multiple linear regression model was performed.

**Results:**

One hundred fifty-six participants completed the survey (response rate: 59.1%). The Cronbach’s alpha for attitude (9 items), subjective norm (6 items), perceived behavioural control (5 items), self-efficacy (6 items), intention (11 items) and past behaviour (15 items) were 0.945, 0.720, 0.751, 0.864, 0.934 and 0.955 respectively. The multiple linear regression analysis found a higher score of the subjective norm (*p* = 0.016), a higher score of self-efficacy (*p* < 0.001), younger age (*p* < 0.001) and having PhD (*p* = 0.038) were associated with increased intention score.

**Conclusions:**

It was shown that higher self efficacy and positive beliefs of their peers and other healthcare professionals were associated with their higher intention score for providing pharmaceutical care. Younger age and having a PhD were other factors associated with their intention to provide pharmaceutical care.

## Background

The importance of postgraduate clinical pharmacy programs has increased with the growth in publications reporting drug-related problems (such as drug-drug interactions, wrong doses or administration routes, and adverse effects) and their consequences. The opportunities for clinical pharmacy practices were first started in the United States (US) in the early 1960s [1]. American College of Clinical Pharmacy (ACCP) defined clinical pharmacy as a health science discipline in which pharmacists provide patient care that optimizes medication therapy and promotes health, wellness, and prevention of disease [2]. Clinical pharmacy education can be carried out theoretically and practically in hospitals, outpatient clinics, and all other centers where healthcare is provided. In addition, after completing specialization education, clinical pharmacists can provide pharmaceutical care services in infectious diseases, oncology, geriatrics, pediatrics, nutrition, etc. [3].

Postgraduate programs aim to introduce the basics of clinical pharmacy and pharmaceutical care, clinical pharmacy research areas, and the research techniques used in clinical pharmacy. Clinical pharmacy education is given within the scope of clinical pharmacy residency training or postgraduate programs in Türkiye. Clinical pharmacy education was started at the undergraduate level at Marmara University in 1991 and at Hacettepe University in 1994. Since 1996 at Marmara University and 2014 at Hacettepe University, postgraduate education has been pronounced. Today, clinical pharmacy graduate programs are carried out in several universities in Türkiye [4]. Clinical pharmacy residency program, which started in 2018 following the adoption of the law on specialization in pharmacy in 2014. This training consists of theoretical courses, case presentations, intensive clinical training, and a specialization thesis for three years. Still, clinical pharmacy services, including pharmaceutical care, do not provide routinely, and the responsibilities of clinical pharmacists are not evident in Türkiye [5].

In countries where clinical pharmacy has been newly introduced, the delivery of pharmaceutical care services is more affected by individual and environmental factors [6]. It is essential to identify barriers and facilitators that affect the behaviour of clinical pharmacists to provide optimal pharmaceutical care, especially after completing postgraduate education programs.

Psychosocial models may help identify critical determinants of individuals’ intentions. Several of these models of behaviour have been used to investigate barriers to the implementation of medical practice or health behaviour change, including the theory of goal-oriented behaviours and the transtheoretical model of behaviour change [7, 8]. Planned Behavior Theory (TPB) is often used to predict behaviour. According to this theory, attitude towards the behaviour, subjective norm, perceived behaviour, and intention influence the emergence of behaviour [9]. To the best of knowledge, there is no Turkish TBP based scale to assess the intention of clinical pharmacists to provide pharmaceutical care.

This study aimed to identify factors associated with the intention of Turkish clinical pharmacists and candidates (who were graduates and students of postgraduate clinical pharmacy programs) to provide pharmaceutical care.

## Methods

### Study type, setting, and subjects

In this prospective observational study, an online survey (prepared by using Marmara University Questionnaire System powered by LimeSurvey©) was sent to all the graduates or students of postgraduate clinical pharmacy programs in Türkiye via e-mail between June 2021- April 2022. Convenience sampling was used. The population of the study consisted of all the graduates or students of postgraduate clinical pharmacy programs (clinical pharmacist residency program, PhD program for individuals with a master of science degree in clinical pharmacy or PhD program for individuals with a bachelor degree of pharmacy, master of science [MSc] with thesis and MSc without thesis) in Türkiye. One reminder email was sent after eight weeks. No incentive was offered to participate in the study. The study was approved by the Hacettepe University Non-Interventional Clinical Trials Ethics Committee (GO21/520). The participants completed an electronical informed consent form.

### Online survey

Online survey was consisted of two parts. First part included participants’ demographic characteristics (including age, gender, postgraduate clinical pharmacy program type, graduate or candidate, and clinical pharmacy program education year).

Second part included TPB based questionnaire. This questionnaire was created using the TPB literature and a manual designed for health services researchers to use when developing TPB questionnaires [6, 10, 11]. The developers granted permission to adapt the Turkish version of this questionnaire. First, the English version of the questionnaire consists of 52 items reflecting attitude (11 items), subjective norm (4 items), perceived behavioural control (5 items), self-efficacy (6 items), intention (11 items) and past behaviour (15 items). The items of attitude (A), subjective norm (SN), perceived behavioural control (PBC), self-efficacy (SE) and intention (INT) were scored on a 5-point Likert scale with possible answers being strongly disagree, disagree, neutral, agree and strongly agree. The past behaviour (PB) scale items were scored on a 5-point Likert scale (never [1] to rarely [2], occasionally [3], often [4] and frequently [5]). Then, the Turkish translation and cultural adaption processes were performed using the International Society for Pharmacoeconomics and Outcomes Research (ISPOR) guideline [12]. Five clinical pharmacy experts evaluated TPB-based questionnaires for content validity. A pilot study was conducted with 20 pharmacists to ensure the clarity of all items. An expert panel evaluated comments from the pilot study and it was revised two items to assess pharmacists’ attitude scale. For test–retest analysis, the questionnaire was applied to another thirty pharmacists (responses were not included in the dataset of the study population) at baseline and two weeks later. Completion of this survey took approximately 15–20 min.

### Sample size

Required sample size is determined by statistical power analysis. This requires the specification of the study design and the expected effect size [13]. It is reasonable to assume at least a moderate effect size (i.e. multiple R of around 0.3) [14] for TPB studies using a multiple regression approach. Generally, a sample size of 80 would be acceptable [15]. In addition, according to Rashidian et al. [16], a sample of more than 148 respondents is deemed sufficient to apply the TPB. This sample size was calculated from the variance inflation factor method (VIF), as proposed by Hsieh et al. [17], based on a correlation of 0.25 between intention and behaviour, and of 0.4 between intention and perceived behavioural control. VIF is calculated as the ratio of the variance of a coefficient in a model with multiple predictors, divided by the variance of that coefficient in a model with only one predictor. This method was considered as more sensitive to the variation in parameters of a TPB study. Sample size was also calculated based on previous research about clinical pharmacy services [6] according to expected correlation coefficient which detect the statistical significance of a correlation of minimum 0.70, with alpha at 0.05 and power of 0.80, the required sample size was calculated as 107. In conclusion, according to these approaches explained at TPB based studies above [15–17], the sample size of this study was ranged from 80 to 148.

### Statistical analysis

Data were evaluated using descriptive statistics (mean, standard deviation, median, interquartile range, frequency and percentages). SPSS version 3 (IBM, Armonk, NY) was used for the statistical analysis in the study. Shapiro–Wilk tests were used to determine the data’s normality. The intraclass correlation coefficient for test–retest reliability was performed. Exploratory factor analysis (EFA) was used with varimax rotation. Kaiser–Meyer–Olkin Measure of Sampling Adequacy and Bartlett’s Test of Sphericity was evaluated. Cronbach’s alpha was calculated for the total scale and each construct of TPB. Univariate analysis was conducted to identify factors associated with providing pharmaceutical care to individuals who are graduates or students of postgraduate clinical pharmacy programs on the TPB. After univariate analysis, variables with *p* < 0.25 were determined as candidate variables. Then the multiple linear regression model was established. *p* < 0.05 was considered as the level of statistical significance.

## Results

Two hundred sixty-four participants accessed the link and 156 participants completed the survey (response rate: 59.1%). The median value of age [IQR] of participants was 34 [12] years. Twenty-point-five percent of the participants were male. The demographic data are given in Table 1.

**Table 1:** The demographic data of participants (*n* = 156)

**Variables**	n (%)
**Gender**	
Female	124 (79.5)
Male	32 (20.5)
**The clinical pharmacy programs**	
MSc without thesis	63 (40.4)
MSc with thesis	93 (59.6)
Clinical pharmacy residency program	23 (14.7)
PhD program for individuals with master of	15 (9.6)
Science degree on clinical pharmacy	13 (8.3)
PhD program for individuals with bachelor degree of pharmacy	12 (7.7)
**Postgraduate clinical pharmacy programs**	
Candidate	57 (36.5)
Graduate	99 (63.5)
**Clinical pharmacy program education year**	
< 5 years	133 (78.8)
≥ 5 years	23 (21.2)

The intraclass correlation coefficient was 0.82 between baseline and within two weeks (*p* < 0.001). Kaiser–Meyer–Olkin measure of sampling adequacy yielded a value of 0.860 and Bartlett’s Test of Sphericity was significant (*p* < 0.001). Explanatory factor analysis determined six subscales which explained 64.9% of the total variance. Cronbach’s alpha for TBP based scale was 0.943. The Cronbach’s alpha for attitude (9 items), subjective norm (6 items), perceived behavioural control (5 items), self-efficacy (6 items), intention (11 items), and past behaviour (15 items) were 0.945, 0.720, 0.751, 0.864, 0.934 and 0.955 respectively. Factor loadings are given in Table 2. Each item has a high and substantial loading (> 0.70) on its underlying construct, indicating convergent validity.

**Table 2:** Factor Loadings

	**A**	**SN**	**PBC**	**SE**	**PB**	**INT**
A1	0.81					
A2	0.86					
A3	0.80					
A4	0.85					
A5	0.86					
A6	0.86					
A7	0.74					
A8	0.71					
A10	0.62					
A11	0.74					
A12	0.66					
SN1		0.72				
SN2		0.70				
SN5		0.43				
SN7		0.42				
PBC1			0.68			
PBC2			0.67			
PBC3			0.62			
PBC4			0.61			
PBC5			0.66			
SE6				0.65		
SE7				0.83		
SE8				0.78		
SE9				0.72		
SE10				0.79		
SE11				0.69		
PB1					0.69	
PB2					0.73	
PB3					0.81	
PB4					0.72	
PB5					0.81	
PB8					0.78	
PB9					0.75	
PB10					0.79	
PB11					0.68	
PB12					0.81	
PB13					0.78	
PB14					0.86	
PB15					0.84	
PB16					0.76	
PB17					0.62	
INT1						0.69
INT2						0.81
INT3						0.74
INT4						0.84
INT5						0.86
INT6						0.73
INT7						0.76
INT8						0.77
INT9						0.73
INT10						0.73
INT11						0.67
***Cronbach alfa***	0.945	0.720	0.751	0.864	0.955	0.934
***Median [IQR]***	53 (6)	15 (3)	19 (3.5)	22 (5)	54 (17)	45.5 (7.5)

*A* Attitude, *SN* Subjective norm, *PBC* Perceived behavioral control, *SE* Self efficacy, *PB* Past behavior, *INT* Intention

Table 3 shows the median (IQR), mean (± sd) and percentages of positive and negative responses to the items in each sub-dimension. In terms of attitudes, almost all participants thought that the pharmaceutical care service provided by clinical pharmacists was effective in preventing medication errors (97.4%), rational use of drugs (97.4%), reducing drug costs (96.2%), increasing the quality of treatment (98.7%), improving treatment results (98.1%), and increasing the quality of life of patients (98.1%).

**Table 3:** Survey statements and descriptive statistics (*n* = 156)

Construct	Survey statement	Median (IQR)	Mean ± SD	Positive/high frequency	Negative/ low frequency
A	Pharmaceutical care services provided by clinical pharmacists assist physicians in preventing medication errors and effectively enhancing medication safety	5 (0)	4.75 ± 0.58	152 (97.4)	4 (2.6)
	Pharmaceutical care services provided by clinical pharmacists supports rational drug use and contributes to optimizing drug efficacy	5 (0)	4.78 ± 0.56	152 (97.4)	4 (2.6)
	Pharmaceutical care services provided by clinical pharmacists can reduce drug costs and efficiently enhances pharmacoeconomic drug use	5 (0)	4.66 ± 0.63	150 (96.2)	6 (3.8)
	Pharmaceutical care services provided by clinical pharmacists can effectively enhance the quality of medical services	5 (0)	4.73 ± 0.59	154 (98.7)	2 (1.3)
	Pharmaceutical care services provided by clinical pharmacists can provide substantial benefit to the patients’ health outcomes	5 (0)	4.69 ± 0.62	153 (98.1)	3 (1.9)
	Pharmaceutical care services provided by clinical pharmacists can enhance patients’ quality of life	5 (0)	4.66 ± 0.63	153 (98.1)	3 (1.9)
	Clinical pharmacists’ providing a pharmaceutical care service would enhance their business success	5 (0)	4.69 ± 0.69	148 (94.9)	8 (5.1)
	Clinical pharmacists’ providing a pharmaceutical care service would enhance their job satisfaction	5 (0)	4.71 ± 0.67	149 (95.5)	7 (4.5)
	Pharmaceutical care services would enhance physicians’ trust in pharmacists	5 (0)	4.45 ± 0.77	144 (92.3)	12 (7.7)
	Pharmaceutical care services would enhance patients’ trust in pharmacists	5 (0)	4.62 ± 0.68	151 (96.8)	5 (3.2)
	Pharmaceutical care services would enhance other healthcare workers’ trust in pharmacists	5 (0)	4.51 ± 0.74	148 (94.9)	8 (5.1)
SN	The physicians I work with agree to my providing a pharmaceutical care service	4 (0)	3.55 ± 0.81	79 (50.6)	77 (49.4)
	The nurses in the hospital I work agree to my providing a pharmaceutical care service	4 (0)	3.80 ± 0.87	108 (69.2)	48 (30.8)
	Hospital pharmacists in the hospital I work support me in providing a pharmaceutical care service	4 (0)	3.81 ± 0.83	101 (64.7)	55 (35.3)
	It is important for me what the physicians or nurses think about the services I provide	4 (1)	4.00 ± 0.81	125 (80.1)	31 (19.9)
PBC	I have the necessary knowledge and skills to provide a pharmaceutical care service	4 (0)	4.01 ± 0.65	130 (83.3)	26 (16.7)
	I have the necessary education to provide a pharmaceutical care service	4 (0)	4.08 ± 0.62	134 (85.9)	22 (14.1)
	Clinical pharmacists have necessary equipment support (such as software) to provide a pharmaceutical care service	4 (0)	3.63 ± 0.92	90 (57.7)	66 (42.3)
	It would be entirely up to me whether or not to provide a pharmaceutical care service	3 (1)	3.17 ± 1.09	70 (44.9)	86 (55.1)
	Whether I am a qualified pharmacist or not would entirely be up to me	4 (0)	3.73 ± 1.01	106 (67.9)	50 (32.1)
SE	It would be difficult for me to collect patient-specific information	3 (1)	3.01 ± 1.04	57 (36.5)	99 (63.5)
	It would be difficult for me to detect patients’ medication-related problems	4 (1)	3.65 ± 0.89	21 (13.5)	135 (86.5)
	It would be difficult for me to prevent patients’ medication-related problems	4 (0)	3.59 ± 0.83	20 (12.8)	136 (87.2)
	It would be difficult for me to solve existing medication-related problems	4 (0)	3.68 ± 0.81	17 (10.9)	139 (89.1)
	It would be difficult for me to monitor outcomes of drug therapy	4 (0)	3.48 ± 0.90	32 (20.5)	124 (79.5)
	It would be difficult for me to record pharmaceutical care activities in a patient file	4 (0)	3.38 ± 1.04	39 (25.0)	117 (75.0)
PB	Perform patient-specific medication review	4 (1)	3.77 ± 0.96	96 (61.5)	60 (38.5)
	Assessing the patient’s medications for potential drug-related problems	4 (1)	3.87 ± 0.95	107 (68.6)	49 (31.4)
	Creating a care plan to achieve treatment goals	4 (1)	3.40 ± 1.09	74 (47.4)	82 (52.6)
	Recording appropriately identified drug-related problems	3 (1)	3.33 ± 1.27	74 (47.4)	82 (52.6)
	Planning appropriate follow-up to evaluate patient-specific outcomes	3 (1)	3.31 ± 1.20	70 (44.9)	86 (55.1)
	Making recommendations for the determination of the appropriate dose	4 (0)	3.63 ± 1.09	91 (58.3)	65 (41.7)
	Making recommendations for the determination of the appropriate dosage form	4 (0)	3.57 ± 1.14	89 (57.1)	67 (42.9)
	Making recommendations to determine the appropriate duration of treatment	4 (0)	3.49 ± 1.10	85 (54.5)	71 (45.5)
	Providing education and counseling to the patient in order to determine and increase the patient’s compliance with the treatment	4 (1)	3.77 ± 1.08	104 (66.7)	52 (33.3)
	Making recommendations to reduce drug incompatibility	4 (1)	3.72 ± 1.11	99 (63.5)	57 (36.5)
	Doing therapeutic drug monitoring	3 (1)	3.15 ± 1.35	72 (46.2)	84 (53.8)
	Making recommendations for the reduction of adverse drug reactions	4 (1)	3.78 ± 1.05	100 (64.1)	56 (35.9)
	Making recommendations to reduce possible drug-drug interactions	4 (1)	3.93 ± 0.97	111 (71.2)	45 (28.8)
	Making recommendations for determining the appropriate time of drug administration	4 (1))	4.04 ± 1.01	124 (79.5)	32 (20.5)
	Designing scientific research on pharmaceutical care	3 (1)	2.84 ± 1.31	46 (29.5)	110 (70.5)
INT	I intend to reduce medication-related problems	4 (1)	4.44 ± 0.62	150 (96.2)	6 (3.8)
	I intend to review patients’ medication orders and files in order to detect and prevent prescription errors	4 (1)	4.26 ± 0.75	138 (88.5)	18 (11.5)
	I intend to take part in therapeutic drug monitoring services if required	4 (1)	4.18 ± 0.82	130 (83.3)	26 (16.7)
	I intend to monitor and enhance patients’ compliance with treatment plan	4 (1)	4.43 ± 0.53	153 (98.1)	3 (1.9)
	I intend to observe effects of drug therapy and patients’ achieving desired outcomes	4 (1)	4.36 ± 0.56	150 (96.2)	6 (3.8)
	I intend to provide patients with counseling for safe and appropriate medication use and to offer patient education	4 (1)	4.48 ± 0.53	154 (98.7)	2 (1.3)
	I intend to provide consultancy service for physicians or nurses about medication	4 (1)	4.11 ± 0.84	124 (79.5)	32 (20.5)
	I intend to attend physicians’ case discussion sessions to make recommendations about drug therapy plans for patients and to share medication-related responsibilities with physicians	4 (1)	4.05 ± 0.91	118 (75.6)	38 (24.4)
	I intend to attend physicians’ consultations to make recommendations about drug therapy plans for patients and to share medication-related responsibilities with physicians	4 (1)	4.04 ± 0.91	119 (76.3)	37 (23.7)
	I intend to record my recommendations about medication-related problems	4 (1)	4.22 ± 0.70	140 (89,7)	16 (10.3)
	I intend to take part in the design (establishing a hypothesis), methodology (exploration, data collection), analysis and manuscript writing stages of a scientific research about pharmaceutical care	4 (1)	3.92 ± 0.99	112 (71.8)	44 (28.2)

*A* Attitude, *SN* Subjective norm, *PBC* Perceived behavioral control, *SE* Self efficacy, *PB* Past behaviour, *INT* Intention

Regarding subjective norm, only 50.6% of participants strongly agreed or agreed that the physician would positively approach the pharmacist to provide pharmaceutical care services. In addition, 69.2% and 64.2% of the participants thought that nurses and hospital pharmacists supported the clinical pharmacist to provide pharmaceutical care in the hospital, respectively. Among them, 80.1% indicated that it is important for them what the physicians or nurses think about the services provided by pharmacists.

When perceived behavioural control was evaluated, 83% and 86% of pharmacists thought they had the necessary knowledge, skills, and training to provide pharmaceutical care. In comparison, only 58% thought they had the necessary hardware support. Also, less than half of pharmacists believed that it would be entirely up to them to provide pharmaceutical care.

In terms of self-efficacy, few participants thought it would be difficult to gather information about patients (36.5%), identify drug-related problems (13.5%), prevent drug-related problems (12.8%), solve existing problems (10.9%), monitor treatment (20.5%), and document it (25%).

When past behaviour is taken into account, less than half of the participants stated that they had previously created a care plan for the patients, recorded the problems they detected and made a follow-up plan to evaluate the results. Fifty-three point eight percent of the participants did not do therapeutic drug monitoring. Only 29.5% of pharmacists stated that they had previously participated in the design (hypothesis development), methodology (research, data collection, etc.), analysis, and writing stages of scientific research related to pharmaceutical care. Regarding past behavior, the most common practice of the participants was controlling drug administration times (79%).

When pharmacists’ intentions regarding pharmaceutical care services were questioned, almost all participants stated that they intend to reduce drug-related problems, monitor and increase compliance, monitor the effectiveness of treatment, and counsel and train patients about medications. The least desired practices related to pharmaceutical care were stated as conducting scientific studies, attending physician consultations, and participating in case discussions.

Multiple linear regression analysis exploring the factors associated with the total intention score are presented in Table 4. Subjective norm (*p* = 0.016), self-efficacy (*p* < 0.001), age (*p* < 0.001), and having a PhD (*p* = 0.038) were associated with the total intention score (Table 4). For each 1 point increase in the subjective norm score, there was a 0.512 point increase in the intention score and for each 1 point increase in the self-efficacy score, there was a 0.541 point increase in intention. For each 1 point increase in age, there was a 0.173 point decrease in the intent to provide pharmaceutical care. The mean of total intention score of pharmacists who have a PhD was 0.5 point more than the others (mean ± std. deviation; PhD:4.5 ± 0.11, others: 4.0 ± 0.05).The adjusted R^2^ value was equal to 0.389, which means that the independent variables explained 38.9% of the variation in intent.

**Table 4:** Factors associated with total intention score, multiple linear regression analysis

	**Unstandardized coefficients**		**Standardized coefficients**			**95.0% Confidence Interval for B**		
**Factors**	B	Std. Error	Beta	t	*p*-values	Lower Bound	Upper Bound	VF
Constant	36.120	6.230		5.798	0.000	23.808	48.432	
Attitude total score	-0.029	0.085	-0.026	-0.338	0.736	-0.196	0.139	1.372
Subjective norm total score	0.512	0.210	0.194	2.438	**0.016**	0.097	0.027	1.504
Past behavior total score	0.065	0.038	0.132	1.716	0.088	-0.010	0.140	1.417
Perceived behavioral control total score	0.049	0.157	0.024	0.313	0.755	-0.261	0.360	1.370
Self-efficacy total score	0.541	0.103	0.357	5.239	**0.000**	0.337	0.746	1.108
Age	-0.173	0.049	-0.243	-3.563	**0.000**	-0.269	-0.077	1.106
Having PhD /other)	-5.434	2.589	-0.309	-2.099	**0.038**	-10.550	-0.317	5.169
Clinical pharmacy program education year < 5 years ≥ 5 years	-2.587	1.735	-0.142	-1.491	0.138	-6.015	0.841	2.167
Student/Graduate	-0.178	0.800	-0.029	-0.223	0.824	-1.760	1.403	4.015

*n* = 156 R = 0.623, *R*^2^ = 0.389 *F* = 10.312 *p* < 0.001

## Discussion

To best of our knowledge, this is first study in Türkiye to evaluate intention to provide pharmaceutical care within the theoretical framework of the TPB to individuals who are graduates or candidates of postgraduate clinical pharmacy programs (including MSc [w/wo thesis] and PhD in clinical pharmacy and clinical pharmacy residency program) in Türkiye. It was shown that higher self-efficacy and positive beliefs of their peers and other healthcare professionals were associated with their higher intention score for providing pharmaceutical care. Younger age and having a PhD were other factors associated with their intention to provide pharmaceutical care.

In the present study, in terms of attitudes, almost all pharmacists thought that the pharmaceutical care provided by clinical pharmacists was effective in preventing medication errors, rational use of drugs, reducing drug costs, increasing the quality of treatment, improving treatment results, and increasing quality of life of patients. However, the results show that attitude did not significantly predict intention to provide pharmaceutical care. This indicates that although pharmacists have favourable attitudes toward pharmaceutical care, they do not apply it adequately for various reasons. Based on the answers to the questions of SN, it may be that a significant portion of the health care professionals (physicians, nurses, and pharmacists) are against clinical pharmacists providing pharmaceutical care. Contrary to our study, some scholars argued that attitudes towards the behaviour are the best predictors of intention in the TPB model [18, 19]. Adeoye et al. found that pharmacists’ attitudes were positively associated with medication therapy management completion rates [20]. In addition, Alshehri et al. reported that pharmacists’ attitudes were positively associated with medication therapy management [21]. These conflicting findings can be related to our study population of pharmacists with high attitudes.

The perceived social pressure to perform or not perform the behaviour in question, referred to as SN, was a significant TPB predictor of intention to provide pharmaceutical care in this study. Other studies indicated that SN was also a significant predictor of intention to provide medication therapy management [22] and asthma counselling [23]. Fleming et al. also found SN to significantly predict intention to utilize a prescription drug monitoring program database [24]. Thus, the role of SN in intention formation may be explained by the perception that providing pharmaceutical care has implications for other influential individuals such as physicians, nurses, and hospital pharmacists.

The findings also show that PBC did not significantly predict the intention to provide pharmaceutical care. Similarly, Gazava et al. not found PBC to be a significant predictor of intention to report serious adverse drug events to the Food and Drug Administration (FDA) [22]. Contrarily, Tai et al. found PBC to significantly predict the intention to provide medication disposal education [25]. Most pharmacists were confident they had the necessary knowledge, skills, and education to provide pharmaceutical care. However, they did not generally concede that providing MTM services was entirely their decision. A previous study reached the same conclusion [21]. The provision of pharmaceutical care services requires collaboration among other healthcare professionals, the availability of a documentation system, access to patient medical records and time to devote to pharmaceutical care services. As a result, even if pharmacists are capable and confident in providing MTM services, they would nonetheless require additional support for its implementation.

Similar to PBC in this study, this PB construct was not a significant predictor of intention to provide pharmaceutical care. One of the reasons could be that most pharmacists had never or rarely practised experiences. Furthermore, the most important implication of this finding is that providing pharmacists who have never been involved in patient-centred clinical services with real-world experiences may significantly increase their intentions to provide pharmaceutical care in the future. Similarly, He et al. PB did not significantly predict the intention to provide core clinical pharmacy services [6]. However, Gavaza et al. indicated that the PB could significantly influence pharmacists’ intention to perform health related behaviours [22].

In this study, SE was a significant TPB predictor of intention to provide pharmaceutical care. Regarding self-efficacy, few pharmacists thought it would be difficult to gather information about patients, identify drug-related problems, prevent them, solve existing problems, monitor treatment and document it. Similar results were found in other study [21].

Demographic data were not found to be associated with intent, except for age and having a PhD. The inverse relationship was found between age and intention to provide pharmaceutical care. These results may be because pharmacists are more enthusiastic when they are young and think they have sufficient knowledge after graduating from the clinical pharmacy program. The fact that having a PhD is associated with intention may be due to the fact that people gain experience and increase their self-confidence to provide pharmaceutical care services. Contrary to our study, Herbert et al. not found pharmacists’ characteristics to be a significant predictor of intention to provide medicare medication therapy management services [26].

This study has certain limitations. First, a limited number of pharmacists participated in the study for the planned period; however, the number of participants achieved the sample size requirement. Second, the response rate was calculated based on participants who accessed the questionnaire using the link. Their interest or positive attitude toward pharmaceutical care would influence the response rate. As a result, there could have been a sample bias. Third, because it did not account for all potentially confounding variables, this study cannot draw causal conclusions between TPB factors and actual behaviour.

## Conclusion

To the best of our knowledge, this is the first study in Türkiye to evaluate intention to provide pharmaceutical care within the theoretical framework of the TPB to individuals who are graduates or candidates of postgraduate clinical pharmacy programs (including MSc [w/wo thesis] and PhD in clinical pharmacy and clinical pharmacy residency program) in Türkiye. It was shown that higher self efficacy and positive beliefs of their peers and other healthcare professionals were associated with their higher intention score for providing pharmaceutical care. Younger age and having a PhD were other factors associated with their intention to provide pharmaceutical care. These factors can be evaluated for developing and restructuring clinical pharmacy postgraduate programs in the future.

## Data Availability

The datasets used and/or analysed during the current study available from the corresponding author on reasonable request.
